# Intraoral Extranodal Natural Killer Cell/T-Cell Lymphoma of the Hard Palate

**DOI:** 10.1155/2023/7243119

**Published:** 2023-10-23

**Authors:** Fatemeh Ahmadi-Motamayel, Fereshteh Najar-Karimi, Fatemeh Abbasi, Arash Dehghan

**Affiliations:** ^1^Dental Implants Research Center and Dental Research Center, Department of Oral Medicine, School of Dentistry, Hamadan University of Medical Sciences, Hamadan, Iran; ^2^Department of Oral and Maxillofacial Medicine, School of Dentistry, Alborz University of Medical Sciences, Karaj, Iran; ^3^Department of Oral Medicine, School of Dentistry, Hamadan University of Medical Sciences, Hamadan, Iran; ^4^Department of Pathology, School of Medicine, Hamedan University of Medical Sciences, Hamedan, Iran

## Abstract

Natural killer cell/T-cell lymphoma (NK/TL) is a rare subtype of non-Hodgkin's lymphoma (NHL) associated with the Epstein-Barr virus (EBV) and requires early detection, which can be very challenging. NK/TL often arises in the nasal cavity and can then spread to the oral cavity, but the case presented here is an unusual presentation of NK/TL in a 66-year-old edentulous male patient who initially presented an intraoral exophytic lesion in the palate that appeared within 3 months. We report the present case to highlight the challenges of reaching an accurate diagnosis given the diverse clinical manifestations of NK/TL.

## 1. Introduction

Lymphoma is an unusual heterogeneous group of malignant diseases with two major forms: Hodgkin's lymphoma (HL) and NHL. Lymphoma is caused by the clonal proliferation of lymphoid cells; therefore, Hodgkin's lymphoma mainly affects the lymph nodes in the lymph reticular system, while extranodal involvement is more common in non-Hodgkin's lymphoma and is about 23–30% [1–3]. The head and neck region is the second most preferred site of extranodal NHL after the gastrointestinal tract, while oral cavity involvement, with an average incidence rate of 3.5%, is rare but remains the second most common malignancy in the oral cavity [4]. Currently, NHL is divided into three subgroups: indolent, aggressive, and highly aggressive, according to the most commonly used World Health Organization (WHO) classification regarding clinical behavior and growth rate. Also, regarding the cellular origin, NHL is generally classified into B lymphocyte (B cell), T lymphocyte (T cell), and natural killer T cell (NKT) types [5]. The diffuse large B-cell lymphoma (DLBCL) is the most common type, comprising approximately 30% of all lymphomas, while the NK/TL subtype is rare and accounts for almost 2% of all lymphomas [6, 7].

Intraoral NHL involvement can be represented as a primary or secondary manifestation of a systemic disease [5]. Generally, clinical manifestations of oral lymphoma often appear as asymptomatic exophytic lesions with an elastic consistency that may be ulcerative or hemorrhagic due to trauma. Bone involvement is rare. Since the lymphoma's oral clinical manifestations can appear similar to other diseases, such as periodontal diseases or other malignancies and reactive oral lesions, it is often difficult to diagnose its oral manifestations, which might delay the diagnosis and treatment of the disease, worsening the prognosis [5, 8]. NK/TL is a rare, aggressive subtype of NHL with an extranodal predilection for the nasal cavity. It has been reported that it can be caused by an EBV infection [6].

The purpose of the present case report is to present oral tumoral and aggressive palatal lesions to emphasize the importance of the diagnosis of intraoral manifestation of NK/TL.

## 2. Case Presentation

This paper reports the case of an edentulous 66-year-old male patient after written informed consent was obtained from the patient, who was referred to the Oral Medicine Department, Faculty of Dentistry, Hamadan University of Medical Sciences, with a complaint of growth on the palate with swallowing difficulties, in November 2022. The patient complained of a gradually growing swelling over the past three months, which had recently become ulcerated on the surface and interfered with using his upper denture. The lesion was tender, with no spontaneous bleeding or paresthesia.

The patient was under medical treatment with theophylline, loratadine, and furosemide because of chronic obstructive pulmonary disease and had a smoking history. No other systemic disease was reported. There was no history of trauma or previous infectious diseases such as hepatitis C or acquired immunodeficiency syndrome (AIDS). The patient also experienced noticeable weight loss, fatigue, nausea, and lethargy over the past two months.

In the extraoral examination, the nasolabial fold was slightly obliterated, and no lymphadenopathy was visible in the extraoral examinations.

**Figure 1:**
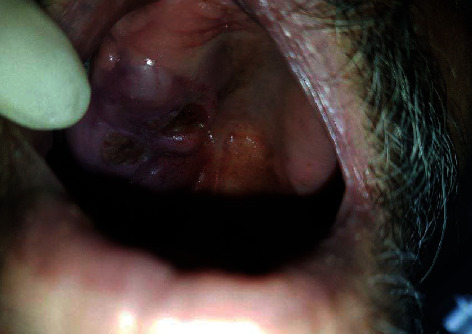
The intraoral manifestation of the lesion.

Intraoral examinations revealed a tumoral exophytic enlargement (sessile) of the lateral posterior right side of the palate with a bosselated surface on the posterior side of the lesion and a firm consistency, except for the middle part of the lesion (near the alveolar ridge), which had a rubbery consistency with a diameter of 3∗4∗5 cm and a slightly blue color. There were two crater-like ulcers measuring 1∗1 cm, covered with a fibrin leukocyte membrane on the posterior aspect of the lesion (Figure 1).

**Figure 2:**
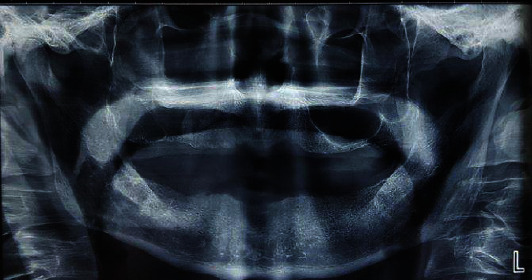
OPG view revealed a radiolucent lesion with irregular borders, filling the right maxillary sinus with erosion and destruction of the sinus's pharyngeal wall. The anterior wall of the pterygomaxillary fissure's integrity has been lost, and the medial wall of the sinus is not visible. To some degree, thinning and erosion in the lower part of the right sinus can be seen.

The panoramic view revealed a radiolucent lesion with irregular borders that filled the right maxillary sinus, along with erosion and destruction of the pharyngeal wall of the sinus. In addition, to some degree, thinning and erosion could be seen in the lower part of the right sinus (Figure 2).

**Figure 3:**
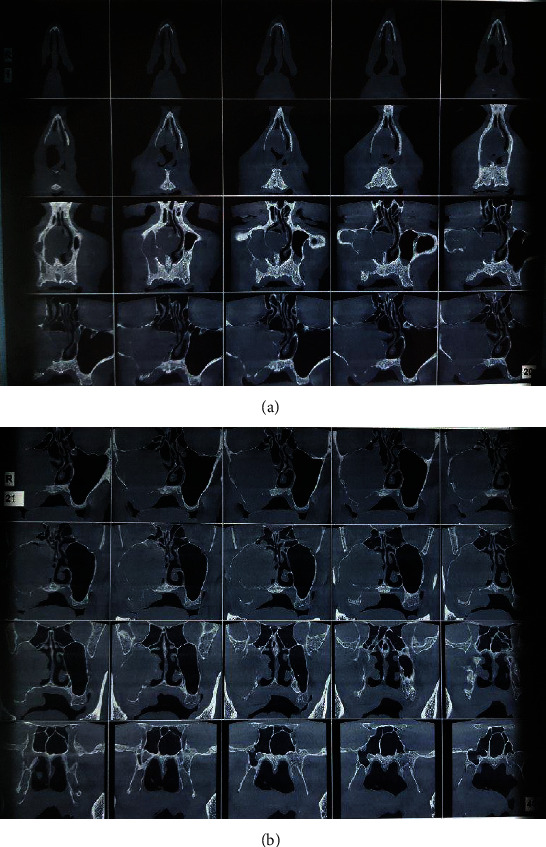
(a, b) Axial sections of cone-beam computed tomography (CBCT) presented a homogeneous hypodense lesion in the right maxillary sinus. The lesion has a destructive nature, destroying the buccal and lingual wall of the right alveolar process. In higher cuts, the lesion has destroyed the sinus's anterior, middle, and posterior lateral walls. The lesion has extended to the nasal cavity by destroying the medial and anterior walls of the sinus and presents as a mass of soft tissue in the nasal fossa. The periosteal reaction is not seen, and the pterygoid processes are intact. The sphenoid sinus is normal without any secretions, but the lateral wall of the right ethmoid sinus has been destroyed by the lesion. The lesion has caused nasal septum deviation.

Cone-beam computed tomography (CBCT) revealed the invasion of the lesion into the maxillary sinus and nasal concha (Figure 3).

Considering the history and clinical manifestations, provisional diagnoses included mucoepidermoid carcinoma (MEC), pleomorphic adenoma (PMA), and acinic cell carcinoma (ACC).

He referred to a private clinic approximately one month ago. The incisional biopsy carried out for him there reported chronic ulcerative squamous mucosa with reactive squamous hyperplasia and obvious dense polymorphic lymphoblastic infiltration in the ulcer bed, which extended to the minor salivary glands.

**Figure 4:**
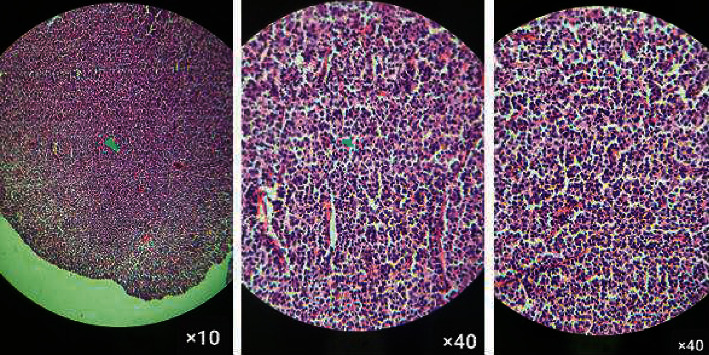
Histopathology findings show a diffuse proliferation of medium-to-large pleomorphic lymphoma cells with regular nuclear membranes, some distinct nucleoli, unclear cells, and cytoplasm borders, which were arranged in dense cell sheets with a fine vascularized and inflammatory background. Much mitotic and apoptotic activity was also observed, with no evidence of specific differentiation.

An incisional biopsy under local anesthesia was carried out, and the sample was sent for routine histopathology examination, which showed neoplastic tissue with a lymphoproliferative nature with the following characteristics: a diffuse proliferation of medium-to-large pleomorphic lymphoma cells with regular nuclear membranes, some distinct nucleoli, unclear cells, and cytoplasmic borders, which were arranged in dense cell sheets with a fine vascularized and inflammatory background. Much mitotic and apoptotic activity was also observed, with no evidence of specific differentiation (Figure 4). These histopathological findings were compatible with a malignant diffuse-type lymphoproliferative disorder that suggested DLBCL. However, there was still a strong emphasis on additional immunohistochemical (IHC) tests to confirm the diagnosis and determine the exact immunotype of the neoplasm.

In the IHC profile, tumor cells were positive for cluster of differentiation 3 (CD3), CD7, CD8, and CD56 but negative for CD4, CD5, CD6, CD10, CD20, Cyclin-D1, and B-cell lymphoma 6 (BCL-6), and the proliferation rate was high with the nuclear protein Ki-67 (Ki − 67) > 60. Therefore, according to IHC findings, a final diagnosis of extranodal NK/TL, nasal type, was confirmed. Also, in situ hybridization was positive for EBV-encoded small RNAs.

Routine blood tests, along with hepatitis C-antibody (HCV-Ab), human immunodeficiency virus-antibody and antigen (HIV-Ab and Ag), and hepatitis B-antigen (HBs-Ag) to rule out AIDS and hepatitis infections, were performed. All the test results were within the normal range, and the results of the infectious tests were negative.

The patient was referred to the Department of Maxillofacial Surgery and Oncology.

If the patient's disease was diagnosed on the first visit and some time did not pass until his second visit and the final diagnosis, the patient would probably have faced fewer treatment challenges.

After being referred to the oncology center, the patient was scheduled to receive 8 chemotherapy sessions according to the CHOP regimen (cyclophosphamide, doxorubicin, vincristine, and prednisone), but after receiving 6 sessions, chemotherapy was discontinued after evaluating the positron emission tomography (PET scan) results, which were satisfactory, due to the occurrence of perianal ulceration and some other complications like reduction of white blood count (WBC). During this period, he was followed up regularly and emphasized about careful oral hygiene. After the completion of the sixth session of chemotherapy, the size of the lesion had noticeably decreased. Then, after consulting the oncologist about the patient's condition, surgery was performed for complete excision of the lesion, and reconstruction of the defective area with prosthetic rehabilitation was performed later. To ensure the absence of any metastases or recurrence, a PET scan was prescribed again approximately 3 months after treatment, and the results were satisfactory.

## 3. Discussion

NK/TL is an aggressive malignancy of natural killer cells or cytotoxic T cells that includes 2% of all T-cell lymphomas. This entity is recognized as a rare, high-grade subtype of NHL (T cells) with a unique extranodal preference for the nasal cavity. EBV infection has been reported to play an important role in this lymphomagenesis process [6]. Asian and South American populations are the most affected by NK/TL; this lesion predominantly involves extranodal sites, especially the nasal cavity. Clinically, primary NK/TL's involvement areas can be classified into two subtypes: a nasal subtype with 80% prevalence, including the nasal cavity, paranasal sinuses, nasooropharynx, and upper respiratory-digestive tract; and a nonnasal subtype with 15–20% prevalence, affecting the skin, gastrointestinal tract, testes, and salivary glands [9]. Also, peripheral blood involvement, called aggressive NK-cell leukemia/lymphoma, is frequently observed [10]. Varying clinical features of extranodal NK/TL make the diagnosis extremely challenging. Nasal-type NK/TL is a lethal midline granuloma that often presents as a destructive tumor in the nasal cavity with upper airway obstruction, epistaxis, facial mass, ulceration, and destruction of the nasofacial area with facial deformities [11].

Although NK/TL often develops in the nasal cavity and then spreads to the mouth, the case described here appeared as an unusual presentation of NK/TL that was initially revealed as an intraoral exophytic lesion of the palate.

Considering the clinical and radiographic features and the patient's history of exophytic and relatively fast-growing lesions (according to the lesion's size during the last three months), and considering the location of the lesion (palate), the ulcerated surface, and its slight blue color, with noticeable weight loss, lethargy, and fatigue history reported by the patient, primary differential diagnoses (in terms of prevalence rate) were salivary gland malignancies, respectively: MEC, PMA, and ACC.

The final diagnosis based on histological and IHC findings with the positive result of EBV detection was extranodal NK/TL, nasal type. In the interpretation of the IHC test, in which antigen-antibody interactions are used to identify specific proteins (in cells), generally, CD3 is a marker of T cells, and CD20 is a marker of B cells, which are expressed in most of mature NK/T lymphomas and mature B-cell lymphomas, respectively. CD56 also plays a role as a NK-cell marker in the diagnosis of NK/T lymphoma. Although not all cases show the same phenotype, the typical IHC profile of extranodal NK/TL is positive for CD2 and cytoplasmic CD3*ε* and CD56, while negative for surface CD3, CD4, CD5, and CD8, along with the presence of cytotoxic molecules (perforin, granzyme B, or TIA1). CD56 is a helpful but nonspecific marker for NK cells, and usually, extranodal NK/TLs are positive for it [6]. In the present case, CD3*ε*, CD7, CD8, and CD56 are positive, and the proliferation rate was high with Ki − 67 > 60.

Although early-stage localized NK/TL is curable with timely diagnosis, patients with more advanced diseases have a poor prognosis [12]. Furthermore, due to the rarity of extranodal NK/TL and the lack of sufficient clinical studies, there are a variety of treatment modalities for NK/TL, and the prognosis depends on the disease stage, location of involvement, and the patient's age [12, 13].

## 4. Conclusion

Extranodal NK/TL is a rare, aggressive lymphoma. We reported the present case as an unusual clinical manifestation of intraoral extranodal NK/TL, highlighting the challenges of reaching an accurate diagnosis, given the diverse clinical manifestations of NK/TL, which can first appear in the oral cavity as an exophytic lesion or ulceration. Therefore, paying attention to the patient's history and performing prompt radiographic and histopathological examinations in particular cases by dentists is essential for early diagnosis and timely treatment to improve the prognosis of malignant lesions.
